# Robust Distance Measures for *k*NN Classification of Cancer Data

**DOI:** 10.1177/1176935120965542

**Published:** 2020-10-13

**Authors:** Rezvan Ehsani, Finn Drabløs

**Affiliations:** 1 Department of Mathematics, University of Zabol, Zabol, Iran; 2 Department of Bioinformatics, University of Zabol, Zabol, Iran; 3 Department of Clinical and Molecular Medicine, NTNU – Norwegian University of Science and Technology, Trondheim, Norway

**Keywords:** *k*-nearest neighbors, *k*NN, distance measures, Fisher, Sobolev

## Abstract

The *k*-Nearest Neighbor (*k*NN) classifier represents a simple and very general approach to classification. Still, the performance of *k*NN classifiers can often compete with more complex machine-learning algorithms. The core of *k*NN depends on a “guilt by association” principle where classification is performed by measuring the similarity between a query and a set of training patterns, often computed as distances. The relative performance of *k*NN classifiers is closely linked to the choice of distance or similarity measure, and it is therefore relevant to investigate the effect of using different distance measures when comparing biomedical data. In this study on classification of cancer data sets, we have used both common and novel distance measures, including the novel distance measures Sobolev and Fisher, and we have evaluated the performance of *k*NN with these distances on 4 cancer data sets of different type. We find that the performance when using the novel distance measures is comparable to the performance with more well-established measures, in particular for the Sobolev distance. We define a robust ranking of all the distance measures according to overall performance. Several distance measures show robust performance in *k*NN over several data sets, in particular the Hassanat, Sobolev, and Manhattan measures. Some of the other measures show good performance on selected data sets but seem to be more sensitive to the nature of the classification data. It is therefore important to benchmark distance measures on similar data prior to classification to identify the most suitable measure in each case.

## Background

Classification and pattern recognition are important challenges in data analysis. The *k*-nearest neighbor (*k*NN) approach was proposed by Fix and Hodges in 1951^1^ and later modified by Cover and Hart in 1967.^2^ It is a simple, robust and versatile algorithm for classification and regression and has been used for different types of problems such as pattern recognition,^3^ ranking of models,^4^ text categorization,^5^ and object recognition,^6^ and in many different areas, including bioinformatics and medicine.^7-9^ It is a non-parametric^10^ and lazy learning classifier. Being non-parametric makes *k*NN free of assumptions on underlying data properties, so there is no need to have prior knowledge about the data. In lazy learning, any generalization of the training data is postponed until the test data are presented to the system.^11^

The *k*NN algorithm is conceptually simple but can still be used on complex biological data, for example, from cancer. A search in the PubMed database for “*k*-NN OR *k*NN” retrieves more than 2000 hits from 1980 to 2020 (August 2020), and a joint search with “cancer” shows that more than 330 of these hits (16%) are related to using the *k*NN approach in cancer research. The popularity of *k*NN actually seems to be increasing; the largest number of hits for both *k*NN itself and the combination of *k*NN and cancer is found in 2019, and for the combination of *k*NN and cancer more than 60% of the hits are found in 2014 or later.

**Figure 1.**
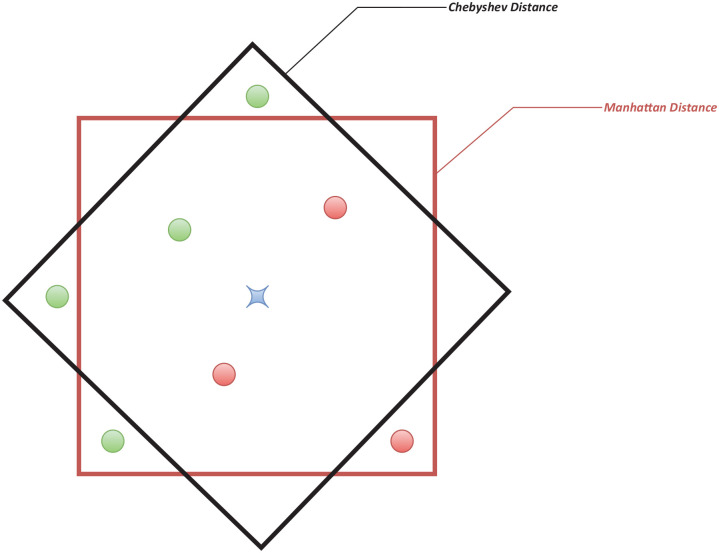
Impact of alternative distance measures on *k*NN performance. Diamond and square shaped neighborhoods are generated by the Chebyshev and Manhattan distances, respectively. In this case, a new query pattern (blue star) would be classified as either green or red by Chebyshev and Manhattan distances, respectively.

The *k*NN algorithm depends upon a neighborhood of close (or similar) patterns relative to a query pattern, and an important challenge is to find the best distance or similarity measure.^12^ Different distance measures will lead to different shapes that define the neighborhood which directly impacts the performance of the *k*NN classifier, as illustrated in Figure 1. However, most applications of *k*NN seem to rely on a limited set of distance measures like Euclidean or Spearman by default, without considering whether alternative distance measures may lead to improved performance.

Several general benchmarking studies have investigated how the performance of the *k*NN algorithm is affected by the choice of distance measure. Chomboon et al^13^ tested the performance of *k*NN with 11 different distance measures including Euclidean, Minkowski, Mahalanobis, Cosine, Manhattan, Chebyshev, Correlation, Hamming, Jaccard, Standardized Euclidean, and Spearman, and they used these distance measures on 8 different binary synthetic data sets. They used cross-validation (70% training and 30% testing) to measure performance and showed that similar accuracy could be obtained using either Manhattan, Minkowski, Chebyshev, Euclidean, Mahalanobis, or Standardized Euclidean, and that these distance measures could outperform several other measures.

Hu et al^14^ evaluated distance measures for *k*NN classification using medical data sets. They focused on 3 different types of data, consisting of categorical, numerical, and mixed data types. The sets were from the UCI repository of data sets for machine learning, and they used 4 different distance measures; Euclidean, cosine, chi-square, and Minkowski. They used cross-validation (70% training and 30% testing) to measure the performances, with *k*-values between 1 and 15. The experiments showed the chi-square distance measure to be best for the 3 different data types, whereas the cosine, Euclidean, and Minkowski distances lead to the lowest accuracy on the mixed-type data set.

Punam and Nitin^15^ used the KDD data set^16^ and the *k*NN classifier with Chebyshev, Euclidean, and Manhattan distance measures. The KDD data set contains numeric data for 41 features in 2 classes. They estimated accuracy, sensitivity, and specificity to evaluate the performance of *k*NN for each distance. The Manhattan distance outperformed the other distances, with 97.8% accuracy, 96.76% sensitivity and 98.35% specificity.

Todeschini et al^17,18^ investigated the *k*NN classifier on 8 benchmark data sets with 18 different distance measures, including Manhattan, Euclidean, Soergel, Lance-Williams, contracted Jaccard-Tanimoto, Bhattacharyya, Lagrange, Mahalanobis, Canberra, Wave-Edge, Clark, Cosine, Correlation, and 4 locally centered Mahalanobis distances. The rate of non-errors and average rank for each distance was determined to evaluate the efficiency of the measure. The results indicated that the highest accuracy was achieved for the Manhattan, Euclidean, Soergel, contracted Jaccard-Tanimoto, and Lance-Williams distance measures.

In a comprehensive review study Prasath and colleagues^19^ investigated the impact of 54 different distance measures on 28 various data sets that were obtained from the UCI machine-learning repository. On most data sets, their work showed the best performance by using the Hassanat distance, compared to the other distances.

In summary, these benchmarking studies (and others) have shown that no distance metric is optimal for all data types. Each data type may require a different distance metric for optimal performance in *k*NN, which is consistent with the principle of “no free lunch.” This makes it relevant to ask how we can guide users with respect to the choice of distance metrics for *k*NN classification of complex data sets to achieve optimal performance. Here, we have tried to answer that question by identifying metrics with relatively consistent performance across a range of complex data sets, using a selection of both common and more novel metrics.

Specifically, we have investigated the performance of *k*NN classification with 12 different distance metrics, including 8 common and well-known metrics (Euclidean, Manhattan, Canberra, Chebyshev, Bray-Curtis, Clark, Hamming, Bhattacharyya), 2 more novel metrics (Hassanat and Soergel), and 2 new metrics presented by us (Sobolev and Fisher). We have tested these metrics on 4 different data sets on cancer; for breast cancer (cytology), brain cancer (imaging), lung cancer (multivariate), and prostate cancer (clinical). We have evaluated the overall performance of each metric by ranking the metrics according to classification performance across these data sets.

## Method

### Data sets

The experiments were done on 4 cancer data sets, for brain, lung, breast, and prostate cancer (see Table 1).

**Table 1.** Summary information for the cancer data sets that were used in this study.

Data set	No. of instances	No. of classes	No. of attributes	Type of data	Reference
**Brain cancer**	3064	3	64*64 matrix	MRI image	[^20^]
**Breast cancer**	699	2	9	Float	[^21^]
**Lung cancer**	32	2	55	Integer	[^21^]
**Prostate cancer**	97	2	9	Float	[^22^]

For brain cancer, we used a data set consisting of 2-dimensional (2D) slices of CE-MRI images for 3 types of tumors; glioma, meningioma, and pituitary tumor. Data for 233 patients with a total of 3,064 images (axial, coronal, and sagittal views) were available. The original size of each image was 512 × 512 pixels, which has been decreased to 64 × 64 to make the calculation faster. The breast and lung cancer data sets were benchmark data sets obtained from the UCI Machine-Learning Repository. The Wisconsin Breast Cancer Data set (WBCD) has 699 instances with 9 attributes for cytology data on 2 types of tumors (i.e. malignant and benign). The lung cancer data set is a multivariate data set with 55 attributes for 32 instances. The prostate cancer data set is a data frame with 97 rows and 9 features with data from a study examining the correlation between the level of prostate-specific antigen and several clinical parameters, using data from participants about to receive a radical prostatectomy.

### Distance measures

Here, we give mathematical formulas for distance measures estimating the closeness between 2 vectors x and y, with $x=(x_{1,\;}x_{2,}.\;\;.\;\;.\;,x_{n})$ and $y=(y_{1,}y_{2,}.\;\;.\;\;.\;,y_{n})$ having numerical attributes. The $d_{m}(x,y)$ is the distance between x and y as measured by m. Formulations and terminologies are mainly taken from Abu Alfeilat et al,^19^ with additional definitions as specified.

#### Minkowski, Euclidean, Manhattan, and Chebyshev distance

This family of distances is defined as:

(1) $$d_{Minkowski}=\sqrt[{p}]{\sum_{i=1}^{n}{\left|x_{i}-y_{i}\right|}^{p}}$$

where p is a positive value. It is the Manhattan distance when p=1, and the Euclidean distance when p=2, whereas the Chebyshev distance is a variant of Minkowski distance where p=∞. This is also known as maximum value distance,^23^ Lagrange,^17^ and chessboard distance,^24^ and can be formulated as:

(2) $$d_{Chebyshev}=\max_{i}\left|x_{i}-y_{i}\right|$$

#### Canberra distance

This weighted version of the Manhattan distance was introduced and later modified by Lance and Williams.^25,26^

(3) $$d_{\mathrm{Canberra}}=\sum_{i=1}^{n}\frac{\left|x_{i}-y_{i}\right|}{\left|x_{i}\right|+\left|y_{i}\right|}$$

#### Hamming distance

This distance is based on the number of differences between 2 vectors.^27^ It is mainly used to analyze nominal data but can also be used for numerical data.

(4) $$d_{\mathrm{Hamming}}=\sum_{i=1}^{n}1_{x_{i}\neq y_{i}}$$

#### Bhattacharyya distance

This distance represents the similarity of 2 probability distributions.^28^

(5) $$d_{\mathrm{Bhattacharyya}}=-ln\sum_{i=1}^{n}\sqrt{x_{i}y_{i}}$$

#### Sorensen distance

This distance is often used to describe relationships in areas like ecology and environmental sciences,^29^ and it is also known as Bray-Curtis. It is a modified Manhattan distance, where the total sum of the values is used to standardize the difference over the vectors *x* and *y*.^30^ It will be between 0 and 1 when all values of the vectors are positive.

(6) $$d_{\mathrm{Bray}-\mathrm{Curtis}}=\frac{\sum_{i=1}^{n}\left|x_{i}-y_{i}\right|}{\sum_{i=1}^{n}\left(x_{i}+y_{i}\right)}$$

#### Clark distance

This distance^31^ is also known as the coefficient of divergence and is the square root of half the divergence distance.

(7) $$d_{\mathrm{Clark}}=\sqrt{\sum_{i=1}^{n}{\left(\frac{x_{i}-y_{i}}{\left|x_{i}\right|+\left|y_{i}\right|}\right)}^{2}}$$

#### Soergel distance

This distance (also known as the Ruzicka distance) is widely used for calculating evolutionary distances.^32^ It is identical to the complement of the Jaccard or Tanimoto similarity coefficient for binary variables,^32^ and it is in accordance with all 4 metric properties provided that all the attributes have non-negative values.^33^

(8) $$d_{\mathrm{Soergel}}=\frac{\sum_{i=1}^{n}\left|x_{i}-y_{i}\right|}{\sum_{i=1}^{n}\max(x_{i},y_{i})}$$

#### Hassanat distance

This non-convex distance was introduced by Hassanat.^34^

$$d_{Hassanat}=\sum_{i=1}^{n}D(x_{i},y_{i})$$

where

(9) $$D\left(x_{i},y_{i}\right)=\left\{ \begin{array}{l} 1-\frac{1+\min\left(x_{i},y_{i}\right)}{1+\max\left(x_{i},y_{i}\right)},\;\;\;\min\left(x_{i},y_{i}\right)\geq 0\\ 1-\frac{\begin{array}{l} 1+\min\left(x_{i},y_{i}\right)+\\ \left|\min\left(x_{i},y_{i}\right)\right| \end{array}}{\begin{array}{l} 1+\max\left(x_{i},y_{i}\right)+\\ \left|\min\left(x_{i},y_{i}\right)\right| \end{array}},\;\;\;\min\left(x_{i},y_{i}\right)<0 \end{array}\right.$$

#### Sobolev distance

Definitions and notations for this distance are as given by Villmann.^35^ Starting with the standard *p*-inner product

(10) $${〈x,y〉}_{p}={\left(\sum_{i=1}^{n}{\left|x_{i}.y_{i}\right|}^{p}\right)}^{\frac{1}{p}}$$

the Sobolev inner product, norm, and metric of degree *k* can be defined as follows:

(11) $${〈x,y〉}_{p,\alpha }^{S}={〈x,y〉}_{p}+\alpha {〈D^{k}x,D^{k}y〉}_{p}$$

(12) $$∥x∥{\;}_{p,k,\alpha }^{S}=\sqrt{{〈x,x〉}_{p,\alpha }^{S}}$$

(13) $$d_{p,k,\alpha }^{S}(x,y)=∥x-y∥{\;}_{p,k,\alpha }^{S}$$

where $D^{k}$ is the *k*th differential operator. There is a connection to the Fourier transform for the special case p=2 and α=1. Let $\hat{x}$ be the Fourier transform *y*

(14) $$\hat{x}(\omega_{k})=\sum_{j=1}^{N-1}y_{j}\exp\left(-i\frac{2\pi kj}{N}\right)$$

where $\omega_{k}=2\pi k/N$ and $i=\sqrt{-1}$. The norm can be defined as

(15) $${\left\|x\right\|}_{2,k,1}^{S}=\sqrt{\sum_{j=1}^{N-1}{\left(1+\omega_{j}\right)}^{k}{\left|\hat{x}(\omega_{j})\right|}^{2}}$$

Here, we have used metric (13) with norm (15) and *k* *=* *1*.

#### Fisher distance

Definitions and notations are as given by Lebanon.^36^ We first define the n-simplex $P_{n}$.

(16) $$P_{n}=\left\{x\in R^{n+1}:\forall i,x_{n}\geq 0,\sum_{i=1}^{n+1}x_{i}=1\right\}$$

The sequence $\left\{x_{i}\right\}$ is the probability of different outputs in each experiment. The Fisher information metric on $P_{n}$ can be defined by

(17) $$J_{ij}=\sum_{k=1}^{n+1}\frac{1}{x_{k}}\frac{\partial x_{k}}{\partial x_{i}}\frac{\partial x_{k}}{\partial x_{j}}$$

The Fisher information is defined as a pull-back metric from the positive *n*-sphere $S_{n}^{+}$;

(18) $$S_{n}^{+}=\left\{x\in R^{n};\forall i,x_{n}\geq 0,\sum_{i=1}^{n+1}x^{2}=1\right\}$$

The transformation $T:P_{n}\to S_{n}^{+}$ defined by

(19) $$T(x)=\left(\sqrt{x_{1}},.\;\;.\;\;.\;,\sqrt{x_{n+1}}\right)$$

pulls back the Euclidean metric on the surface of the sphere to the Fisher information on $P_{n}$. Now Fisher metric for $x,y\in P_{n}$ can be defined as the length of the great circle (geodesic) between T(x) and T(y) on $S_{n}^{+}$.

(20) $$d\left(x,y\right)=\mathrm{acos}\left(\sum_{i=1}^{n+1}\sqrt{x_{i}y_{i}}\right)$$

### Performance measures

We used 4 complementary measures for evaluating the performance of each classifier; accuracy, precision, recall, and *F*_1_. These measures can be computed from the following classifications results, where a subset of patterns (the positive set) belongs to a specific class, whereas the remaining patterns (the negative set) do not belong to this class:

- True positive (TP): The number of patterns of the positive set that is correctly classified as belonging to the positive set.
- True negative (TN): The number of patterns outside of the positive set that are correctly classified as not belonging to the positive set.
- False positive (FP): The number of patterns of the negative set that is incorrectly classified as belonging to the positive set.
- False negative (FN): The number of patterns of the positive set that is incorrectly classified as not belonging to the positive set.

The relevant performance measures can then be defined as:

(21) $$precision=\frac{\mathrm{TP}}{\mathrm{TP}+\mathrm{FP}}$$

(22) $$recall=\frac{\mathrm{TP}}{\mathrm{TP}+\mathrm{FN}}$$

(23) $$F_{1}=2\frac{precision\times recall}{precision+recall}$$

(24) $$accuracy=\frac{\mathrm{TP}+\mathrm{TN}}{\mathrm{TP}+\mathrm{TN}+\mathrm{FP}+\mathrm{FN}}$$

### Ranking of distance measures

For each distance and performance score, we considered the best (maximum) score among scores across all different k∈K values as the final score. If $S_{dpe}^{k}$ is the score of distance d for performance p and experiment e, the final score can be defined as:

(25) Sdpe=Skmaxdpek

We then ranked distances according the final score for each individual experiment, using 2 different approaches. The first approach was simply to compute the average of the ranks across all experiments. That is, for a given experiment e and a given performance measure p, the score S was computed for each distance metric d, and the distance metrics were ranked according to the score. This was repeated for each combination of e and p, giving e×p rankings in total. The final ranking was then estimated as the average ranking of distance metric d over all these e×p rankings. For the second approach, we used RankAggreg tool,^37^ an *R* package for weighted rank aggregation, and we used it on the complete set of ranked lists as described above, using the Cross Entropy Monte Carlo (CE) method, Kendall distances, and a value of rho as 0.1 (please see the RankAggreg documentation).

In addition to the ranking, we used the *k*-means algorithm to cluster the distance measures based on the scores over all experiments, and plotted this using the factoextra^38^ tool in *R*. This highlights in a visual way the similarities and differences between the tested distance measures.

### Software implementation

The Python programming language (version 3.7.1) was used for scripts, which were implemented under Anaconda3. We used libraries from the scikit-learn package (version 0.20.1) to apply the *k*NN algorithm for Euclidean, Manhattan, Chebyshev, Hamming, Canberra, and Bray-Curtis distances.

## Results

We applied all 12 distance measures on the 4 cancer data sets. For the brain, breast, and prostate cancer data sets, we used ranges from 1 up to 20 for *k*. For the lung cancer data, the range of *k* was limited to values from 1 up to 11, due to more limited data.

The best scores for the brain cancer data are shown in Table 2. The best precision score is for Canberra followed by Sobolev and Hassanat. For recall the maximum is shared between Manhattan and Hamming. Second and third places are for Sobolev and Hassanat. The best performances based on *F*_1_ and accuracy were for Canberra and Hassanat, respectively.

**Table 2.** Best scores among all tested *k*-values for the brain cancer data set.

Distance	Precision	Recall	*F*_1_	Accuracy
**Fisher**	0.442	0.387	0.378	0.487
**Sobolev**	0.462	0.460	0.441	0.526
**Clark**	0.435	0.414	0.404	0.493
**Bhattacharyya**	0.434	0.398	0.394	0.492
**Soergel**	0.450	0.444	0.428	0.518
**Hassanat**	0.462	0.460	0.443	**0.529**
**Euclidean**	0.461	0.455	0.432	0.522
**Manhattan**	0.458	**0.461**	0.440	0.524
**Chebyshev**	0.452	0.455	0.438	0.521
**Hamming**	0.445	**0.461**	0.432	0.520
**Canberra**	**0.466**	0.457	**0.448**	0.527
**Bray-Curtis**	0.450	0.445	0.428	0.518

Maximum scores for each performance measure are shown in bold.

The scores for the breast cancer data are shown in Table 3. The Clark distance achieved the best score for 3 performance measures: recall, *F*_1_, and accuracy. The best precision was for the Bray-Curtis distance.

**Table 3.** Best scores among all tested *k*-values for the breast cancer data set.

Distance	Precision	Recall	F_1_	Accuracy
**Fisher**	0.896	0.896	0.892	0.903
**Sobolev**	0.964	0.958	0.960	0.964
**Clark**	0.967	**0.972**	**0.969**	**0.971**
**Bhattacharyya**	0.905	0.895	0.896	0.907
**Soergel**	0.964	0.966	0.964	0.967
**Hassanat**	0.963	0.966	0.964	0.967
**Euclidean**	0.966	0.962	0.963	0.967
**Manhattan**	0.963	0.954	0.957	0.962
**Chebyshev**	0.963	0.961	0.960	0.964
**Hamming**	0.950	0.925	0.934	0.943
**Canberra**	0.966	0.969	0.967	0.970
**Bray-Curtis**	**0.969**	0.969	0.968	**0.971**

Maximum scores for each performance measure are shown in bold.

For the lung cancer data, the Sobolev distance outperformed the other distances, as it had the best performance according to precision, *F*_1_, and accuracy. The second rank was for Fisher distance, which achieved the best score for recall and shared *F*_1_ with Sobolev.

Finally, for the prostate cancer data, the Canberra distance clearly outperformed the other distances according to all performance measures.

To have a total and robust ranking scale we used 2 approaches as described under Methods: a basic average of ranks for each distance measure estimated over 16 different rankings (i.e. all possible combinations of data set and performance measure), and the weighted rank aggregation of these rankings by the RankAggreg tool.

**Figure 2.**
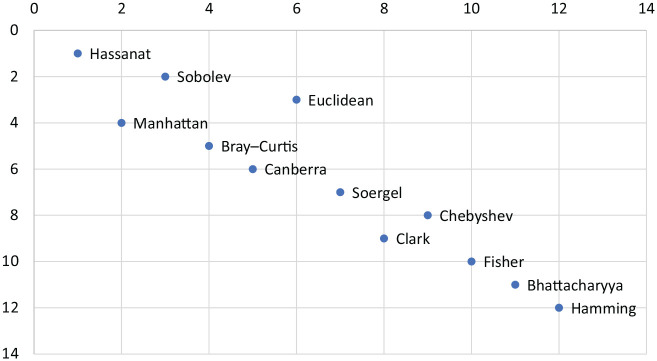
Comparison of average ranking and RankAggreg ranking.

To compare the ranking of these 2 approaches, we plotted the 2 rankings, as shown in Figure 2. This shows a good correlation between these rankings, indicating that the overall ranking of the distance measures is robust.

**Figure 3.**
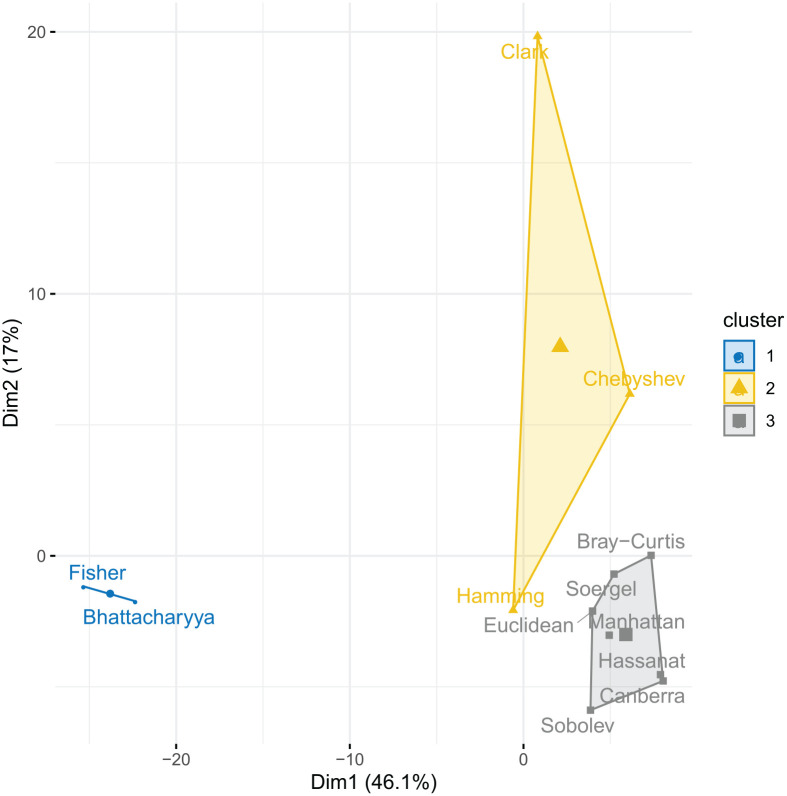
The *k*-means clustering of all scores over all experiments. Axes represent variance in a PCA plot of the data.

The result of the *k*-means clustering on the performance scores over all experiments for *k* = 3 are shown in Figure 3. The set of (Hassanat, Canberra, Sobolev, Manhattan, Euclidean, Soergel, Bray-Curtis) forms a relatively tight cluster, whereas the 2 additional clusters are (Hamming, Chebyshev, Clark) and (Bhattacharyya, Fisher). This is quite consistent with the ranking in Figure 2, where the main cluster is seen to consist of the measures with the best overall performance. A clustering with *k* = 4 splits the main cluster into 2 subclusters consisting of (Sobolev, Manhattan, Euclidean) and (Hassanat, Canberra, Soergel, Bray-Curtis), but the general clustering is stable. In summary, the *k*-means clustering confirms the ranking of the performance data shown in Figure 2.

## Discussion

The results presented here show clear differences between distance measures with respect to classification performance on the cancer data sets. Some distance measures have a quite robust performance across most data sets, whereas other measures show a clearly lower performance on some data sets. This seems to be largely independent of which performance measures that are used (precision, recall, F_1_ or accuracy), which seems to be confirmed by the loading plot of a principal component analysis (PCA) of the performance data from Tables 2 to 5 (Supplemental Figure S2 in Additional file 1). The plot shows very similar loadings for all performance measures for each data set, in particular for the data on breast cancer and lung cancer.

**Table 4.** Best scores among all tested *k*-values for the lung cancer data set.

Distance	Precision	Recall	*F*_1_	Accuracy
**Fisher**	0.611	**0.656**	**0.602**	0.613
**Sobolev**	**0.650**	0.633	**0.602**	**0.618**
**Clark**	0.144	0.356	0.198	0.307
**Bhattacharyya**	0.553	0.600	0.511	0.545
**Soergel**	0.550	0.578	0.522	0.545
**Hassanat**	0.489	0.544	0.464	0.516
**Euclidean**	0.617	0.633	0.582	0.590
**Manhattan**	0.618	0.611	0.582	0.585
**Chebyshev**	0.268	0.389	0.262	0.351
**Hamming**	0.449	0.500	0.418	0.459
**Canberra**	0.584	0.544	0.503	0.507
**Bray-Curtis**	0.550	0.578	0.522	0.545

Maximum scores for each performance measure are shown in bold.

**Table 5.** Best scores among all tested *k*-values for the prostate cancer data set.

Distance	Precision	Recall	*F*_1_	Accuracy
**Fisher**	0.868	0.792	0.769	0.813
**Sobolev**	0.856	0.832	0.823	0.832
**Clark**	0.892	0.836	0.840	0.861
**Bhattacharyya**	0.864	0.783	0.755	0.803
**Soergel**	0.836	0.820	0.812	0.822
**Hassanat**	0.864	0.835	0.829	0.841
**Euclidean**	0.856	0.845	0.834	0.841
**Manhattan**	0.846	0.828	0.821	0.832
**Chebyshev**	0.864	0.845	0.834	0.841
**Hamming**	0.625	0.625	0.594	0.598
**Canberra**	**0.926**	**0.882**	**0.877**	**0.892**
**Bray-Curtis**	0.836	0.820	0.812	0.822

Maximum scores for each performance measure are shown in bold.

The individual classification results in Tables 2 to 6 show important differences (and similarities) between the distance measures, depending on data type. If we focus on the *F*_1_ performance measure, we see that both Fisher and Bhattacharyya seem to have relatively low performance on brain cancer (Table 2), breast cancer (Table 3), and prostate cancer (Table 5), in addition to Hamming for prostate cancer. This is different for lung cancer (Table 4), where it is Clark and Chebyshev that is associated with low performance. These differences seem to be confirmed by the *k*-means clustering (Figure 3), where both (Fisher, Bhattacharyya) and (Clark, Chebyshev, Hamming) form separate clusters, and by the PCA analysis, where the loadings for breast cancer data are clearly separated from the other cancer types (Supplemental Figure S2 in Additional file 1). It is also consistent with the ranking data shown in Figure 2, where these same distance measures are ranked together as having low performance.

**Table 6.** Total ranking of distance measures over all experiments according average rank and rank aggregation by RankAggreg.

Distance	Average rank	Rank_Ave	RankAggreg
**Hassanat**	3.50	1	1
**Manhattan**	4.18	2	4
**Sobolev**	4.56	3	2
**Canberra**	4.64	5	6
**Bray-Curtis**	4.62	4	5
**Euclidean**	4.81	6	3
**Soergel**	5.43	7	7
**Clark**	5.56	8	9
**Chebyshev**	5.68	9	8
**Fisher**	5.87	10	10
**Bhattacharyya**	6.06	11	11
**Hamming**	6.68	12	12

The ranking of the well-performing measures shows some variation, but this is mainly due to the generally good performance of these measures, with only small (and partly random) differences between cases. However, it is important to realize that the performance of a given distance measure depends on the input data. For example, in the data on lung cancer (Table 4) the Fisher measure shows one of the best performances, whereas it shows low performance on the other data sets. Similarly, the Clark measure is the best-performing measure on breast cancer data (Table 3) but has very low performance on lung cancer data. Apart from intrinsic effects of the type and distribution of data, these differences could arise from the distance functions, which is something that is relevant for further studies.

The analysis presented here may be influenced by the quality of the input data, for example, whether cases in the training set are correctly annotated with respect to class (e.g. cancer versus normal). In principle, we can estimate the quality of training data by looking for consistent misclassifications, experiments where a case consistently is classified to a different class compared to its annotation. Such cases may represent potential annotation errors in the data set and may be considered for removal. However, we should probably expect to have some examples of such cases in most data sets consisting of experimental data. In particular for complex properties like cancer, where it may be difficult to decide unambiguously in each case whether a given sample should represent “cancer” or “normal.” In the data presented here, the somewhat lower classification performance on brain cancer data and lung cancer data can possibly be linked partly to misannotated cases. However, such cases will be a natural part of most experimental data and removing them may introduce user bias into the analysis. Also, *k*NN is supposed to be somewhat robust with respect to errors in training data, in particular for higher values of *k*, as the classification will represent an average over multiple cases. Therefore, we have not considered removing such cases from the analysis.

This analysis will also be influenced by the choice of features, for example, if we select only specific features for analysis, compared to the full range of features of a data set. This may for example be relevant if the features represent very different properties. Again, selecting subsets of features may introduce user bias into the analysis. Here, we wanted to test the robustness of the various distance metrics, and therefore, we decided to use all features as given in the original data sets, without any feature selection.

## Conclusions

The performance analysis of *k*NN classification of cancer data with different distance measures identifies important differences between both distance measures and data sets. It is possible to identify a subset of distance measures that show robust performance across several data sets, and this includes the Hassanat, Sobolev, and Manhattan measures. However, the study also confirms that no single distance measure will be optimal for all data sets, and the recommendation must be that several measures should be tested on suitable reference data that are as similar to the actual data as possible when selecting distance measure for a particular study.

## Supplemental Material

Suuplemental_figure – Supplemental material for Robust Distance Measures for *k*NN Classification of Cancer Data Click here for additional data file. Supplemental material, Suuplemental_figure for Robust Distance Measures for *k*NN Classification of Cancer Data by Rezvan Ehsani and Finn Drabløs in Cancer Informatics
