# Supplementary material for: Robust Distance Measures for kNN Classification of Cancer Data
Source: Cancer Inform. 2020 Oct 13;19:1176935120965542. doi: 10.1177/1176935120965542 (PMC7573750; doi:10.1177/1176935120965542)
Supplement: Suuplemental_figure – Supplemental material for Robust Distance Measures for kNN Classification of Cancer Data [file Suuplemental_figure.pdf]

PCA on score values for classification performance

The score values for classification performance as listed in Table 2-5 of the main paper (Rezvan & Drabløs, 2020) were merged into a single table and used for a principal component analysis (PCA), using the web resource ClustVis <https://biit.cs.ut.ee/clustvis/>. Unit variance scaling was applied to rows, and SVD with imputation was used to calculate principal components. The result is shown in Fig. S1. X and Y axis show principal components 1 and 2 that explain 46.1% and 27.6% of the total variance, respectively, for N = 12 data points (*i.e.*, distance measures). The PCA loadings are shown in Fig. S2.

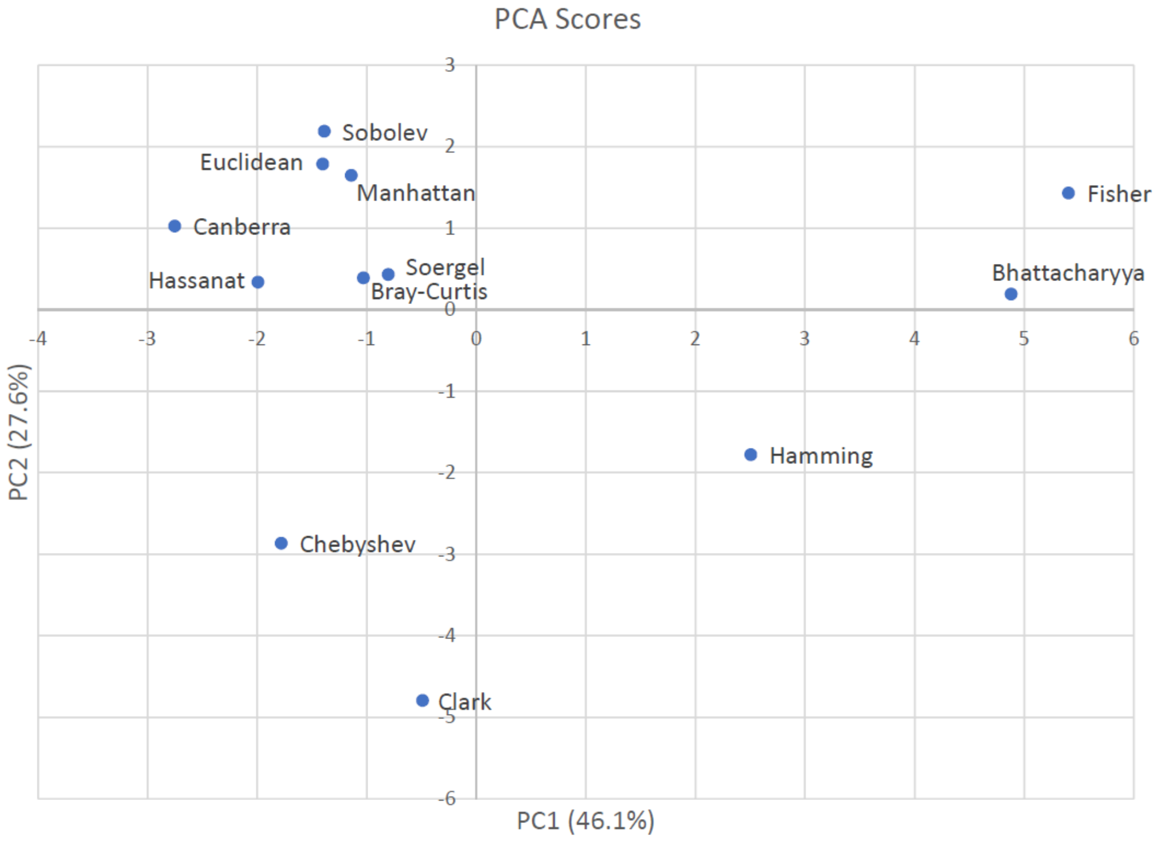

Fig. S1 PCA scores for classification performance. The first two components explain 46.1 and 27.6% of the variance, respectively.

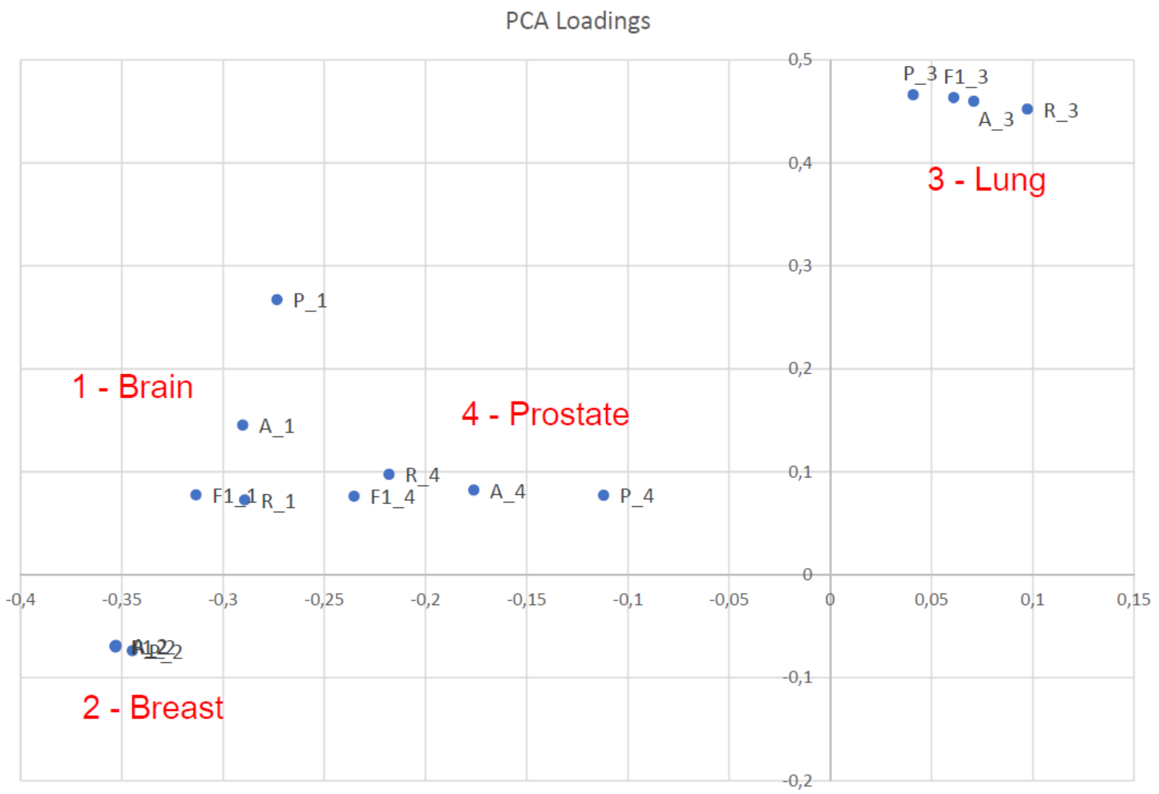

Fig. S2 PCA loadings for classification performance. The data points represent loadings for each data type (1 – Brain cancer; 2 – Breast cancer; 3 – Lung cancer; 4 – Prostate cancer) and for each performance score type (P – Precision; R – Recall; F1; A – Accuracy).
